# Phenological responses of 215 moth species to interannual climate variation in the Pacific Northwest from 1895 through 2013

**DOI:** 10.1371/journal.pone.0202850

**Published:** 2018-09-12

**Authors:** Julie A. Maurer, Jon H. Shepard, Lars G. Crabo, Paul C. Hammond, Richard S. Zack, Merrill A. Peterson

**Affiliations:** 1 Biology Department, Western Washington University, Bellingham, Washington, United States of America; 2 Oregon State Arthropod Collection, Oregon State University, Corvallis, Oregon, United States of America; 3 Department of Entomology, Washington State University, Pullman, Washington, United States of America; Universite du Quebec a Chicoutimi, CANADA

## Abstract

Climate change has caused shifts in the phenology and distributions of many species but comparing responses across species is challenged by inconsistencies in the methodology and taxonomic and temporal scope of individual studies. Natural history collections offer a rich source of data for examining phenological shifts for a large number of species. We paired specimen records from Pacific Northwest insect collections to climate data to analyze the responses of 215 moth species to interannual climate variation over a period of 119 years (1895–2013) during which average annual temperatures have increased in the region. We quantified the effects of late winter/early spring temperatures, averaged annually across the region, on dates of occurrence of adults, taking into account the effects of elevation, latitude, and longitude. We assessed whether species-specific phenological responses varied with adult flight season and larval diet breadth. Collection dates were significantly earlier in warmer years for 36.3% of moth species, and later for 3.7%. Species exhibited an average phenological advance of 1.9 days/°C, but species-specific shifts ranged from an advance of 10.3 days/°C to a delay of 10.6 days/°C. More spring-flying species shifted their phenology than summer- or fall-flying species. These responses did not vary among groups defined by larval diet breadth. The highly variable phenological responses to climate change in Pacific Northwest moths agree with other studies on Lepidoptera and suggest that it will remain difficult to accurately forecast which species and ecological interactions are most likely to be affected by climate change. Our results also underscore the value of natural history collections as windows into long-term ecological trends.

## Introduction

Recent climate change has caused shifts in the phenology and distribution of many species [1–3]. By decoupling trophic interactions and pushing species to the limits of their geographic distributions, these shifts pose a serious threat to biodiversity and to the integrity of ecosystems [1, 4–5]. Numerous studies spanning an array of spatial and temporal scales, using a variety of methodological approaches, and focusing on numerous different taxa, have made it clear that species-specific responses to climate change are far from uniform [3–4, 6]. Unfortunately, the diversity in methodology and duration of these studies has challenged our ability to make meaningful comparisons of the responses of different species to climate change and to assess whether those responses vary predictably among geographic locations, taxonomic groups, or life history traits [3, 7–8].

Natural history specimen records and past faunistic and floristic surveys offer valuable archives of species-specific phenologies and distributions, which could supplement data gathered from long-term monitoring schemes and short-term studies. Compared to long-term monitoring studies [2, 7, 9], collection records generally provide a deeper wealth of historical data for studying how these patterns have shifted under the accelerated climate change of the industrial era, and to what degree these shifts vary among taxa [3, 7, 10]. However, such data sources are fraught with all manner of analytical challenges; collectors of natural history specimens are biased in which taxa they sample, when, where, and how intensively they sample, and they often retain only the specimens in the best condition [8, 10–11]. Independent of collector biases and climate change effects, species phenology may change in association with distributional shifts in response to land-use changes. Furthermore, abundance may increase or decrease in response to climate change, land use change, or other factors such as introduced species [12], which may alter the point in the flight period of a particular species at which it is sufficiently abundant to have a high likelihood of detection. These biases make it difficult to determine whether phenological shifts are due to climate change or changing patterns of spatiotemporal sampling effort or changes in population distribution. In many ways, such challenges are similar to those presented by data from citizen science initiatives such as iNaturalist [13], BugGuide [14], and others, in which citizen scientists post what are often idiosyncratically gathered natural history observations to a centralized server [15]. Nonetheless, enabled by recent statistical advances (e.g., generalized linear mixed effects modeling), it is possible to increase the signal to noise ratio in such unconventional data sources [7, 11, 16], opening the wealth of data in natural history collections and citizen science projects for use in untangling the complex responses of organisms to climate change [3, 7, 15].

Species occurrences in the Lepidoptera have been chronicled by natural historians for well over a century, so butterfly and moth collections contain a trove of useful data for examining among-species variation in responses to climate change [6]. Recent studies have shown that many Lepidoptera species are indeed sensitive to climate change, exhibiting distributional or phenological shifts in conjunction with changing climate on both local and broad scales [7–9]. Furthermore, these responses vary among species and depend to some degree on life history traits such as adult seasonality, overwintering stage, and larval diet [17–18]. For example, species that overwinter in developmentally advanced stages (pupae) and tend to fly in spring generally exhibit stronger phenological responses to temperature variation than do species that overwinter in less advanced stages (eggs or larvae) and fly in summer or fall [2, 19–20]. Such patterns are presumably due to the combined effects of cues that trigger the termination of insect diapause and completion of development (e.g., temperature, photoperiod, precipitation) [21], as well as how much post-winter development is needed to achieve adulthood (reviewed in [22–23]). Larval diet also influences phenological responses to climate change in moths and butterflies, in that species that feed on woody plants have stronger responses than those that feed on herbaceous plants [20], and specialists show stronger shifts than generalists [19]. These effects of diet breadth may relate to the underlying need to synchronize larval development with seasonal shifts in the defensive chemistry of woody plants or with the seasonally-varying availability of a specific plant species [17].

In this study, we analyzed a natural history collection specimen database documenting the occurrences of 215 functionally diverse moth species over 119 years (1895–2013) in British Columbia, Washington, Oregon, Idaho, and western Montana, a large region in western North America often referred to as the Pacific Northwest (PNW). Studies examining regional climate change over the 20^th^ century have found that average annual temperatures in the region have increased by 0.6° to 0.9°C, with the shift varying both spatially and seasonally [24]. The most pronounced temperature increases during this time have been in the winter months (December through February), resulting in longer growing seasons [24], Even with the long-term change in regional average temperatures, there has been considerable interannual temperature variation stemming from factors such as El Niño-Southern Oscillation and the Pacific North American pattern [24].

The approach we adopted was a simplification of a fine-grained analysis used by Kharouba and colleagues [8] to examine the phenological signature of climate change on collection dates for Canadian butterflies, in which each collection record was associated with temperature profiles from the nearest weather station for the year of capture. Challenges with this approach are that finding the nearest weather station for each collection event is a labor-intensive process, data from weather stations are often incomplete, and collections made in remote areas may not have a sufficiently nearby station for comparison. For these reasons, we adopted a more streamlined, but coarser-grained approach, assessing whether capture dates of moths vary with interannual variation in temperature profiles averaged across a large geographic region. Our primary goal was to examine the responses of each species to climatic variation, focusing on the effect of temperature on the capture dates of moth specimens, and whether those effects varied among life history categories based on adult seasonal phenology and larval diet breadth. Our reasoning for focusing on temperature was that a) previous studies have shown phenological shifts in the Lepidoptera in response to temperature [2, 8, 25], b) there is a clear link between temperature and the termination of diapause and developmental rates in moths and other insects [17, 22–23], and c) climate change models have generally found temperature shifts are easier to predict than changes in precipitation [24] We hypothesized that moths would have earlier capture dates in warmer years, and that the degree of phenological shift would be greatest for spring-eclosing species and larval dietary specialists, based on results that others have obtained in analyzing the effects of climate change on Lepidoptera communities [2, 19, 26]. A secondary goal of our study was to provide a simplified analytical approach that others could use to analyze natural history specimen data for evidence of the phenological effects of climate change. Finally, our study highlights the value of the continued collection of natural history data.

## Materials and methods

### Moth occurrence & temperature data

The data used in this study were drawn from the Pacific Northwest Moths database [27], a repository of > 90,000 georeferenced occurrence records for more than 1,200 moth species representing all ‘macromoth’ families in the region except the Geometridae. As defined for this study, an occurrence record could be from specimen data across the PNW region (the vast majority), but could also be records from the literature, records from collections outside the region, or photo-vouchered records collected by citizen scientists. Each occurrence record in the database includes the location (and associated latitude, longitude, and often the locality elevation) for one or more specimens of a species sampled on a given date at that location. For any collection localities for which the elevation was missing, we used the Elevation Lookup Utility function of GPS Visualizer [28], which uses GPS coordinates to generate an approximate elevation.

Data from the Pacific Northwest Moths database were exported on October 8, 2015 and the database was subsequently reduced to ensure a consistent and accurate assessment of species-specific phenological responses to interannual climate variation. We first removed all records that were outside of the PNW region. Subsequently, based on notes associated with individual records, we removed all records for specimens raised from immature stages, because rearing conditions were likely to have resulted in atypical adult emergence dates. We further reduced the dataset by removing all records with incomplete dates (missing day, month or year of capture). To remove the complications associated with assessing the phenological responses of multi-brooded species, we then screened the remaining species for evidence of tightly unimodal phenologies and retained only the records for such species. This included removing species with broad, but unimodal phenologies, the great majority of which are likely multivoltine species with overlapping broods. We also removed univoltine species that overwinter as adults because temperatures experienced over two growing seasons likely shape their phenologies. Subsequently, to reduce bias and/or error due to small sample sizes, we removed all moth species with fewer than 75 complete collection records in the database. Multiple records of a species from a locality in a given year were reduced to the median Julian Date (JD) of capture across those records to avoid pseudoreplication. We used median flight date instead of first flight date because first flight date can exhibit strong bias due to among-year, among-site, or among-taxon differences in collection intensity [8]. After data reduction based on all of these criteria for excluding species and/or records, the database included 32,058 occurrence records representing 215 moth species. When possible, we assigned species to functional group categories based on adult seasonality and larval diet breadth (Table 1). Species with inadequate larval data and species with broad phenological peaks overlapping with more than one category were not assigned functional group memberships for those categories but were still included in the main analysis (See S1 and S2 Tables for more information).

**Table 1:** Moth functional groups as defined for this study.

Functional Group Category	Description
*Adult Seasonality*	
Early Season	Unimodal phenology; peak between start of year and June 10.
Mid Season	Unimodal phenology; peak between June 11 and July 31.
Late Season	Unimodal phenology; peak between July 31 and end of year.
*Larval Diet Breadth*	
Monophagous	Larvae feed on plants in one genus.
Oligophagous	Larvae feed on plants in multiple genera in one family.
Polyphagous	Larvae feed on plants in multiple families.

For each year with moth collection records (almost every year from 1895 to 2015), we pulled regional temperature data from the Climate at a Glance database hosted by the U.S. National Center for Environmental Information [29]. Specifically, we obtained the average February through April temperature across the entire western region, as defined by the U.S. National Weather Service (NWS), an area that includes Arizona, California, Nevada, Utah, Idaho, Oregon, and Washington. We used this average as a proxy for the PNW region, which, as defined for this study includes British Columbia, Washington, Oregon, Idaho, and western Montana, because we know of no database that maintains annual temperature averages for the specific geographic area covered by our study. We chose the three-month window of February through April because late winter and spring temperatures can greatly influence the timing of insect development in temperate regions due their effect on both the termination of diapause and the rate of development following diapause [19, 22–23]. Furthermore, using temperatures for a fixed period of months, rather than having a sliding window that depends on the seasonality of the species, enables us to compare our results with others who have used such an approach (e.g., [2, 8, 19]). We converted each region-wide three-month temperature average to temperature anomalies from the norm by subtracting the average February-April temperature for a given year from the average for that period across all years in which we had species occurrence data.

### Analysis

**Fig 1:**
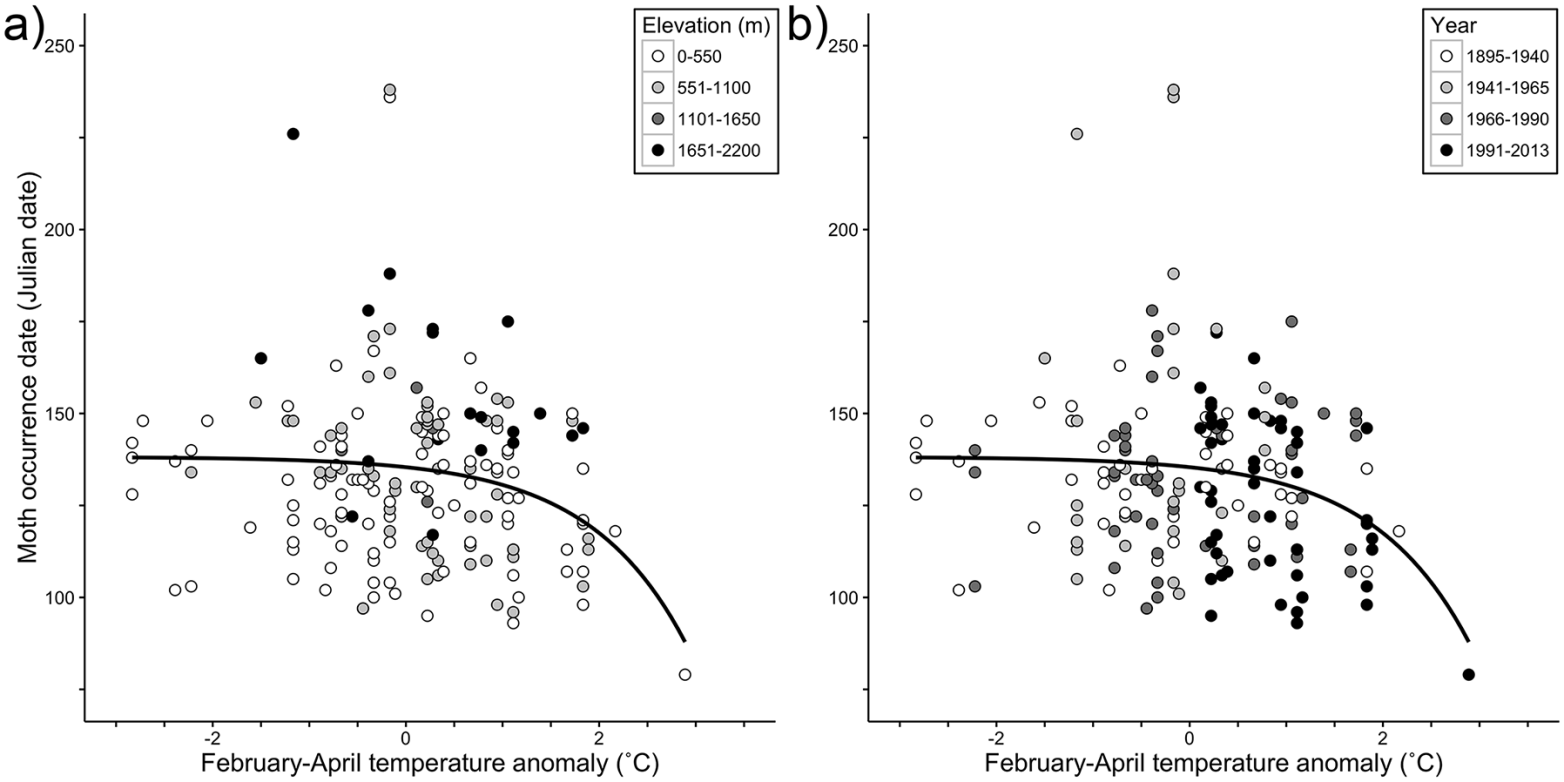
Response curve of date of capture for an early-season specialist, *Leptarctia californiae* (Walker, 1855), to regional February-April temperature anomalies. The response curve was calculated with the GLMM model slope and intercept of the relationship between temperature anomaly and log_e_(date of capture), but it is plotted using a linear Y-axis for ease of interpretation. Negative February-April anomalies represent colder than average years while positive values represent warmer than average years. (a) Response curve plotted with elevation. Elevation was included as a random effect in our GLMM because higher elevation species tend to fly later in the season, explaining some variation in model slope. (b) Collection years plotted with response curve to show changes in phenological response through past 119 years.

We used generalized linear mixed effects models (GLMMs) to determine the effect of yearly February-April temperature anomalies on Julian Date of capture for each species, and to assess whether those effects differed among functional groups defined by adult seasonality and larval diet breadth. Our expectation was that moths would be captured earlier in warmer years, and that this would be evidenced by a negative relationship between temperature anomaly and Julian Date of capture. The GLMM models reduced statistical noise caused by other variables that likely influence date of capture (elevation, latitude, longitude) by treating these variables as random effects. Thus, the results of these analyses should not be skewed by false signals of phenological shifts stemming from temporal changes in the distribution of a species or in where collectors have tended to sample. To determine the best choice of distribution and link functions for our analyses, we performed a GLMM model comparison using the six possible combinations of link functions (log and identity) and distribution functions (Poisson, Gaussian, and quasi-Poisson) on four test species (*Grammia ornata* (Packard, 1864), *Hemaris thetis* (Boisduval, 1855), *Tolype distincta* French, 1890, *Euxoa messoria* (Harris 1841)) that were well represented in the database and featured both spring and late summer specialists. The GLMM model with a Poisson distribution and log_e_ link function had the lowest AIC value for three of these test case species and performed nearly as well as the best model for the fourth species, so we applied this model to all of the other species in our dataset (see Fig 1 for a representative phenology-anomaly relationship). That this combination of distribution and link functions emerged as the best-performing combination is not surprising; each occurrence record is assumed to be independent of the others as is typical in a Poisson distribution [30] and a log_e_ link function is often the preferred link function in GLMMs when the response variable fits a Poisson distribution [31].

Our primary goal in using GLMMs was to determine the phenological sensitivity (days/°C) of each species to interannual temperature variation. We quantified phenological sensitivity by both the direction (earlier vs. later) and magnitude (number of days) of the phenological shift of a species in response to a 1°C increase in February-April temperature. For each species, we determined the direction of that shift from the slope of the relationship between February-April temperature anomalies and log_e_(Julian date of capture). Thus, to test the hypothesis that moths are generally caught earlier in years with warmer February-April temperatures than the historical norm, we used a one-tailed t-test to assess whether, across all 215 moth species, such slopes are significantly less than zero. We determined the magnitude of phenological sensitivity (days/°C) for each species by multiplying a) the slope of the temperature anomaly (°C) vs. log_e_(Julian date of capture) relationship for that species by b) the intercept of that relationship (i.e., the date of capture of that species under an anomaly value of 0). Because the slope of the anomaly vs. log_e_(Julian date of capture) relationship indicates the percentage of change in date of capture, a late-summer species would thus exhibit a higher sensitivity than an early-spring species with an identical slope.

Because of the effect of spring vs. summer flight on the magnitude of phenological sensitivity, we restricted our comparisons across functional groups to comparisons of model slopes (not sensitivities). Specifically, we used ANOVA to determine if slopes differed among functional groups defined by seasonality (early, mid, late) or by larval diet breadth (monophagous, oligophagous, polyphagous) (Table 1). When functional groups differed, we used Tukey HSD to make post-hoc pairwise comparisons between specific functional categories. We also used Bonferroni-adjusted G-tests with post-hoc pairwise comparisons to assess whether functional groups differ in the probability of exhibiting significantly earlier occurrences with increasing temperature anomaly. In addition, we used ANOVA to determine if model slopes varied among species with the number of occurrences (sample size) for those species. Analyses comparing model slopes across functional groups and sample sizes did not include adjustments for phylogenetic non-independence because we were unable to obtain a well-resolved phylogeny for the entire suite of 215 species. MtDNA sequence data are available via Barcode of Life Data Systems [32] for the great majority of species in this study, but for many of the species, such sequence reads were either incomplete or entirely lacking. Our conclusions regarding the effects of life history traits on responses to climate change should thus be viewed as provisional, though that would arguably be the case even if we had an mtDNA-based phylogeny for our full set of species, given the fact that the evolutionary history of mtDNA is expected to not be representative of the history of the entire genome [33]. That said, it is notable that recent studies examining the effect of climate change on Lepidopteran phenology have found that phylogenetically adjusted models provide qualitatively similar results to models without such adjustments [8, 19].

## Results

**Fig 2:**
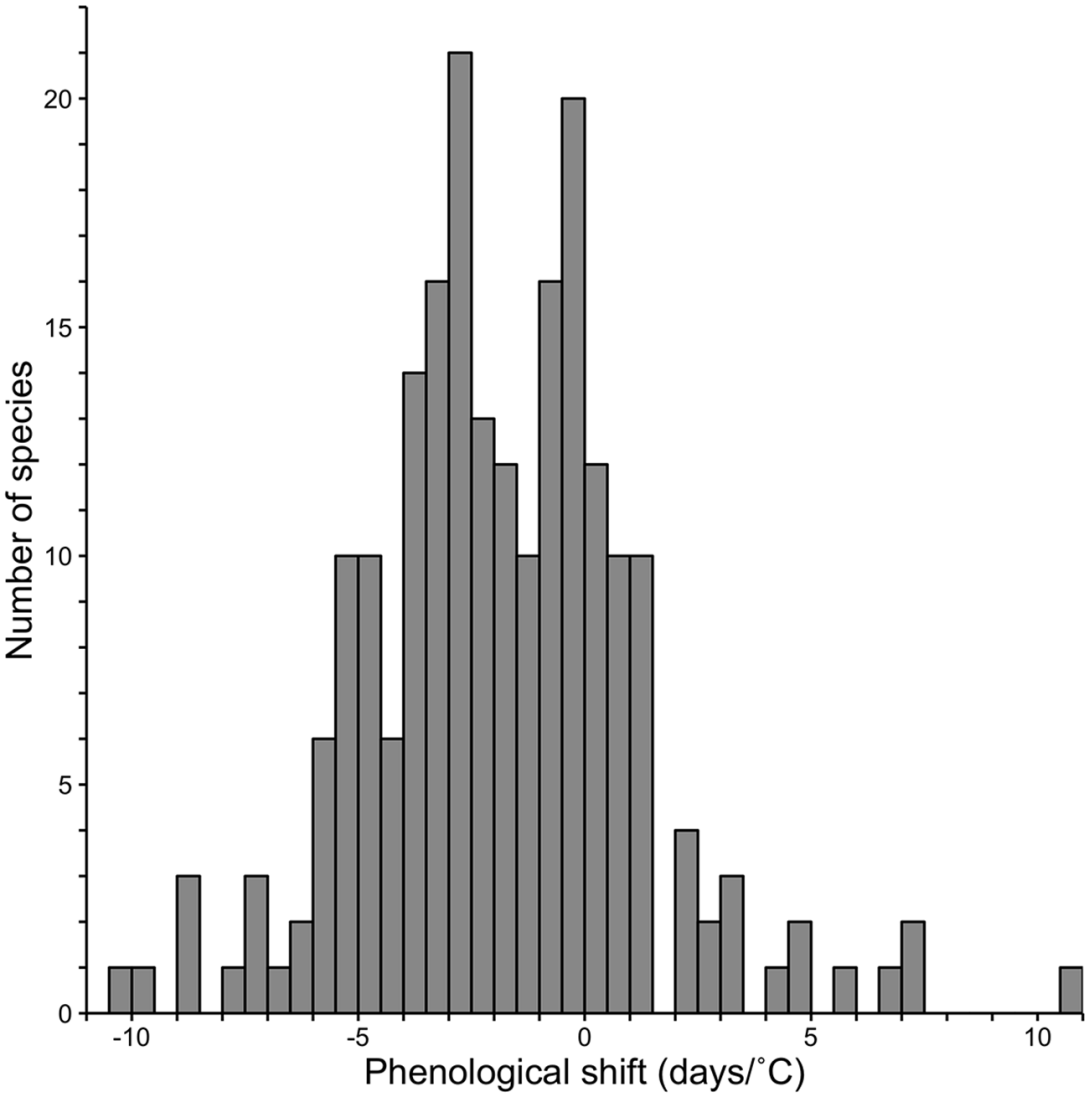
Frequency distribution of phenological sensitivities (days/°C) of moth species to regional February-April temperature anomalies. Zero indicates no shift in flight date, negative values indicate earlier capture dates and positive values indicate later capture dates in years with a 1°C deviation from zero. Mean sensitivity is 1.9 days/°C across all 215 species.

We found that, on average, moth species in the PNW have been collected significantly earlier in years with warmer February-April regional average temperatures. Specifically, the average slope of the anomaly vs. log_e_(date of capture) was significantly negative (*t*_214df_ = -9.07, *P* < 0.001). Furthermore, 36.3% of the species had significantly earlier capture dates in warmer years (i.e., a significant negative slope for the Julian Date vs. temperature anomaly relationship), whereas only 3.7% of the species were captured significantly later in warmer years; the remainder (60%) did not exhibit a significant phenological shift in response to temperature change. Sample size (i.e., the number of observations for a species) did not have a significant effect on the slope of the anomaly vs. log_e_(date of capture) relationship (*F*_1,213_ = 0.072, *P* = 0.79). There was considerable variation among moth species in the magnitude of their phenological sensitivity to among-year differences in February-April temperatures. Indeed, species exhibited shifts ranging from 10.3 days earlier in response to a 1°C increase in the regional temperature to as much as 10.6 days later for every 1 °C increase (mean sensitivity = -1.9 days/°C, Fig 2; see S3 Table for species-specific values). For example, *Leptarctia californiae* which clearly occurred later at higher elevations under a given temperature anomaly (Fig 1a), exhibited an average shift of -3.5 days/°C. This species also illustrates that earlier flight dates tended to occur in recent years (Fig 1b), with exceptions that included 1934, the warmest year on record for the region [24].

**Fig 3:**
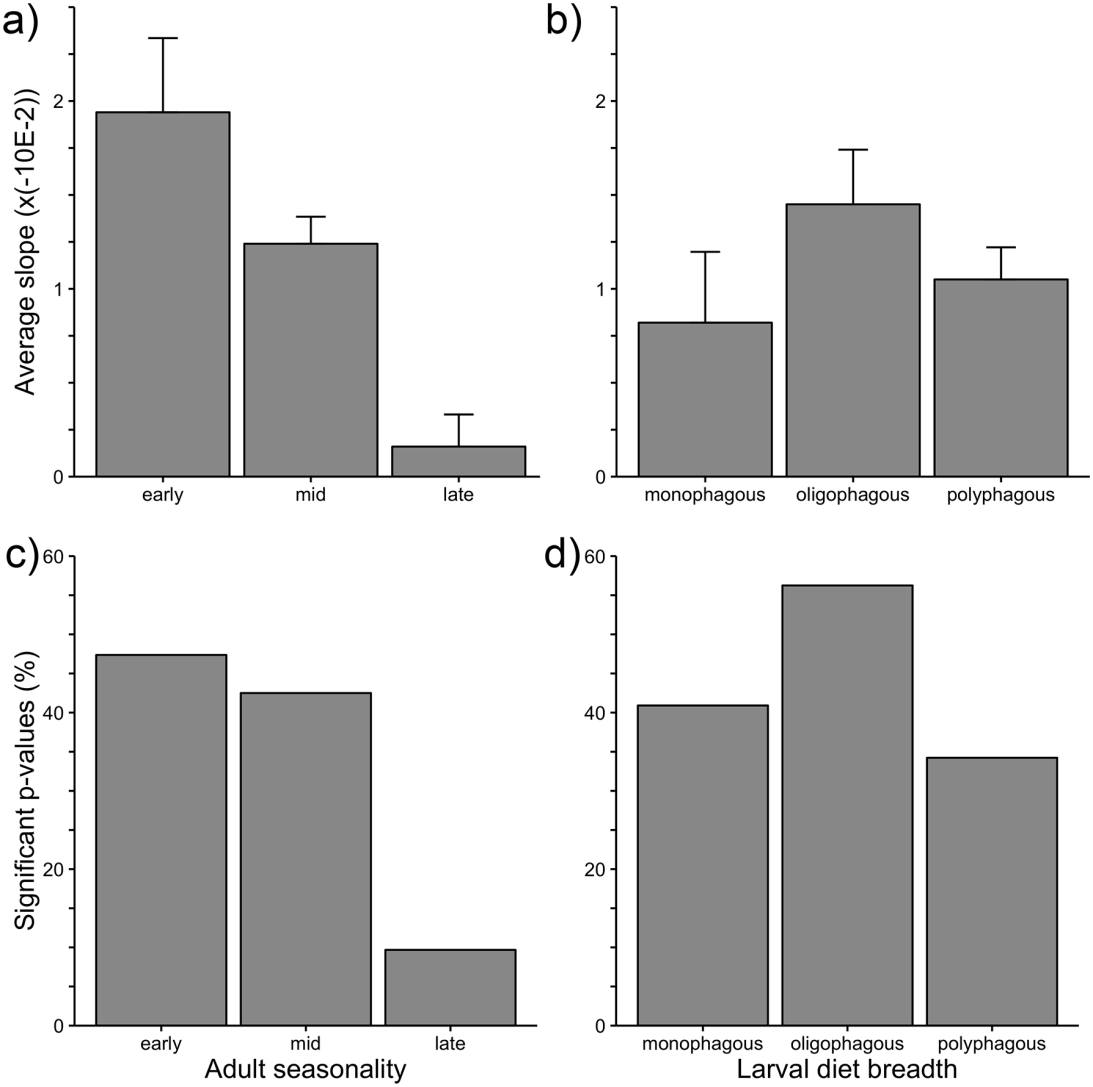
Comparisons of model slopes among phenological and dietary functional groups. (a,b) Comparing average model slope for the relationship between temperature anomaly and log_e_(date of capture) (inverted for ease of interpretation). (c,d) Comparing percent of significant slopes for the percentage of species flying significantly earlier in warmer years. Significant pairwise differences are indicated by differences in letters above the bars. See Methods for explanation of seasonality and diet breadth categories.

For the 210 (out of 215 total) species we could assign to adult seasonality categories, there were 36 early-season specialists, 123 mid-season specialists, and 51 late-season specialists. For the 178 species for which we could determine larval diet breadth, 21 species were monophagous, 46 species were oligophagous, and 111 species were polyphagous. The response of moth species to variation in regional February-April temperatures varied significantly among functional groups based on adult seasonality (*F*_2,207_ = 13.2, *P* < 0.001, Fig 3a), with early-season specialists having a much stronger shift in flight date (indicated by a steeper anomaly vs. log_e_(date of capture) relationship) than mid-season or late-season specialists. In addition, early-season species were more likely to have a significantly negative slope than their later-flying counterparts (*G*_2 df_ = 14.9, *P* < 0.001, Fig 3c). Functional groups based on larval diet breadth showed no difference in slope steepness (*F*_3,211_ = 0.00036, *P* = 0.32, Fig 3b). Furthermore, although the likelihood of having a significantly negative slope varied among monophagous, oligophagous, and polyphagous species in an overall analysis (*G*_2 df_ = 47.6, *P* < 0.001, Fig 3d), none of the pairwise comparisons among these categories differed in this regard.

## Discussion

### Climate change and phenology

The primary purpose of this study was to quantify climate-related shifts in phenology for a large assemblage of PNW moth species, using data from natural history collection specimens sampled over a period spanning more than a century. As predicted, moths collectively have earlier dates of occurrence in years with warmer temperatures in late winter to early spring, with the average capture date shifting by nearly two days per 1°C increase above historical average temperatures. However, both the magnitude and direction of this response varied considerably among species, ranging in extremes of phenological shifts from 10.3 days earlier to 10.6 days later per 1°C increase. Climate projections for the PNW suggest that these shifts may substantially increase with future climate change; compared to an average temperature increase in the region of 0.6 °C to 0.9 °C over the last century [24], the rate of warming is projected to increase to 0.1 to 0.6 °C per decade over the next 100 years, based on IPCC emissions scenarios [34].

The average phenological sensitivity of PNW moths in response to climate change corresponds well with estimates for other Lepidoptera, as does the amount of species-specific variation in such sensitivity. For example, an analysis of collection data for 204 species of Canadian butterflies estimated that dates of occurrence had advanced by an average of 2.4 days/°C earlier over a period of 138 years, but the estimates for individual species ranged from 10.1 days earlier to 13.0 days later per 1°C increase [8]. Average dates of collection for a suite of 187 butterfly species in British Columbia were advanced between 1.5 and 3.8 days/°C over a period of 133 years, with a species-specific range from approximately 14 days earlier to 4 days later per degree [35]. Similarly, for 33 univoltine species of butterflies collected in the UK over a 91-year period, collection dates had advanced by an average of 4.4 days/°C (range from 8.7 days earlier to 2.1 days later per degree) [36]. The broad picture emerging from these studies is that many species of Lepidoptera in temperate regions are shifting to earlier flight dates as temperatures increase, but that the magnitude and direction of phenological shifts are highly variable among species.

The reasons for such pronounced variation in phenological sensitivities estimated from our study and other similar studies are likely to be both biological and methodological in origin. Biologically, the timing of adult flight depends on when diapause is terminated, how long it takes to complete development following diapause, and when adult eclosion is initiated, and these processes are influenced by factors such as temperature, length of the chilling period, photoperiod, and precipitation, and their species- specific interactions [12, 22–23]. Thus, analyses that focus only on temperature will fail to explain the phenological variation of many species. Furthermore, phenological plasticity to temperature may vary among species, with the most plastic species displaying the clearest phenological shifts in response to interannual temperature variation [6]. Another possible biological explanation is that if lowland populations of some species have been disproportionately extirpated due to land use changes, introduced species, or climate change, it might appear that such species are flying later in more recent, warmer years. Our analyses would not be affected by such range shifts, because our models assessed the effects of temperature after taking into account the effects of latitude, longitude, and elevation on phenology.

Methodologically, because natural history specimens are sampled haphazardly across both time and space it is likely that such sparse sampling reduces the accuracy and enhances the variance in estimates of phenological sensitivities [16]. In part, this is because estimates of phenological sensitivities using such data provide a measure of shifts over a large area, which should not be confused with the population-level responses for a given species, which are likely to be stronger and less variable. The results of Roy and colleagues [37], who analyzed weekly counts of 31 butterfly species obtained via standardized citizen science observations that were made annually throughout the growing season at multiple sites over a period of 37 years, illustrate this point. Taking into account the effect of population, the species all exhibited earlier flight dates with increasing temperatures (mean sensitivity = 6.4 days earlier per degree, with a range from 3.7 to 9.1 days earlier per degree). However, when combining data across populations, the mean sensitivity across species was reduced to 4.3 days earlier per degree, and the range was increased to 12.9 days earlier to 3.7 days later per degree. This difference may be due to variation among population-level responses that results from local adaptation and/or spatial variation in temperature or other cues (e.g., photoperiod and/or precipitation) that may influence adult emergence dates [37]. Whatever the mechanism, this result would suggest that the phenological signal from natural history collection data might underestimate the magnitude and overestimate the variance of phenological shifts at the population level, unlike long-term monitoring studies at sentinel sites. Indeed, with rare exceptions [21] the results from such long-term surveys have found that the great majority of species fly earlier in warmer years [19, 25–26]. Thus, it is possible that, had we restricted our analyses to frequently collected localities, phenological sensitivities would have varied less among species, and our estimate of the average phenological shift would have been higher.

### Life history traits and phenological sensitivity

Our finding that spring-flying moth species are more sensitive to interannual variation in late winter temperature than are summer- and fall-flying species is consistent with what several other studies of the phenological responses of moths, butterflies, and other insects have shown [2, 19]. Unlike summer- and fall-flying moths, spring-flying species often overwinter as pupae that are very close to completing development, likely explaining their greater sensitivity to late-winter temperatures [19]. Furthermore, the adult emergence of summer and fall-flying species may depend more on day length and precipitation patterns [2, 12, 22]. Future studies using the PNW moth database should assess whether interannual temperature variation during other seasons (e.g., summer) and variation in precipitation have a disproportionately stronger effect on species that fly late in the growing season. Such analyses were beyond the scope of the present study. Although we were unable to determine if the differential responses of spring, summer, and fall moth species were influenced by phylogenetic non-independence, others (e.g. [8, 19]) have shown that these patterns are not diminished after factoring in the phylogenetic relatedness of taxa.

There was no evidence that the sensitivity of PNW moth species to interannual temperature variation is influenced by larval diet breadth, as demonstrated by Kharouba and colleagues [8] for Canadian butterflies. In contrast, other studies of moths and butterflies have shown that dietary specialists can be either more sensitive to climate change than generalists [19] or less sensitive [36]. Such inconsistency is also evident among studies assessing whether phenological responses to climate change differ for Lepidopteran larvae that specialize on herbaceous vs. woody host plants. For example, Altermatt [38] found that specialists on woody plants exhibit greater phenological sensitivity to climate change than their counterparts on herbaceous plants, while Végvári and colleagues [21] documented the opposite pattern. Thus, it appears that larval diet is a less consistent predictor of the species-specific responses of Lepidoptera than is the season of adult emergence.

### Implications for ecological interactions

A frequently stated concern regarding climate change is its potential to alter ecological interactions in both natural and managed ecosystems via shifts in phenology and/or distribution [1, 18, 39]. These concerns are supported by studies showing that the strength of ecological interactions such as competition, herbivory, predation, pollination, and parasitism can be altered as a result of the differential responses of interacting species to climate change [35, 40–41]. The consequences of such ecological decoupling can be profound, as these shifts can alter fitness, simplify food webs, and even cause local extinction [41]. Although some have argued that the likelihood of such impacts of climate change have been overstated [42], a recent meta-analysis found that the synchrony of interacting species in both terrestrial and aquatic systems has shifted over the period of rapid global warming that has occurred in the past 35 years [5]. However, that study also showed that the magnitude and direction of such changes have been far from uniform, challenging our ability to predict which ecosystems, communities, and species are most likely to be affected by future climate change.

Lepidopterans interact with a wide array of host and nectar plants, competitors, and natural enemies, and climate change may differentially affect the phenologies of these interacting taxa [12, 35, 43]. For example, a comparison of the phenological sensitivities of British Columbia butterflies and their nectar plants, drawing from insect collection and herbarium records, showed that although both groups of organisms had phenological advances under warmer temperatures, the phenologies of flowering plants were, on average, more sensitive to temperature variation than were the phenologies of butterflies. However, the phenological sensitivities of interacting pairs of butterfly and nectar plant species were not correlated [35], illustrating the challenges in predicting which butterfly species are most likely to be impacted by phenological mismatches with nectar plants. It can also be difficult to predict whether interactions with larval host plants may be decoupled, because the degree of synchrony under varying temperatures for a given butterfly species and its host plants can even depend on latitude [43]. Our discovery of extensive variation among moth species in their responses to interannual temperature in the PNW further underscores the fact that accurate modeling of the effects of climate change on ecological interactions involving lepidopterans and the species with which they interact is likely to remain exceptionally difficult.

### Analyzing data from natural history collections

Natural history collections offer valuable archives of data for understanding the ecological impacts of climate change [10]. Furthermore, the availability of such data continues to grow as collections digitize their holdings and post them to online databases via aggregators such as GBIF [44] or via more specialized, region-, taxon-, or question-specific projects [27, 45]. In many ways, repositories of natural history observations by citizen scientists (e.g., BugGuide [14] and iNaturalist [13]) provide data that are similar to data from natural history collections: observations are haphazardly made, sampling is sparse for a given taxon in a given region and is subject to the idiosyncratic biases of individual observers [15]. Despite these shortcomings, data from both natural history collections and citizen science repositories are proving to be useful for studies of the impacts of climate change on both distribution [46] and phenology [8, 36, 42]. In this paper, we show that even a coarse-grained analysis of such data, based on temperatures averaged across a large geographic region, can reveal clear evidence of phenological shifts in response to climate change, as well as a window into the degree of interspecific variation in such responses.

## Conclusions

This study analyzed the phenological responses of moths to interannual temperature variation in the PNW region of the U.S. and adjacent areas in Canada over a period from 1895 through 2013. Using a relatively streamlined analytical approach and drawing from a large database of natural history collection records for 215 moth species, we found that, on average, moths have flown earlier in years with warmer late winter (February) to early spring (April) temperatures. Most of the warmest years for the region have occurred in the last few decades, and the area is predicted to experience even greater warming over the coming decades. Thus, the average phenological advance of 1.9 days/°C seen across the moths in our study suggests that future phenological shifts in the region could be substantial. Our study also documented considerable variation in both the direction and magnitude of phenological shifts exhibited by different moth species, even within functional groups defined by adult seasonality and larval diet breadth. This variation highlights the tremendous challenges we face if we are to predict accurately how ecological interactions involving any given species may be altered under future climate change. Future efforts to better understand the factors underlying the highly variable phenological responses of moth species to temperature variation may benefit by considering different temporal windows of temperature variation, tailored to the seasonality of each species, as well as by considering the effects of interannual variation in precipitation on moth phenology.

## Supporting information

S1 Table Species with multi-functional group membership. All species were classified as having a "mid season" phenology for GLMM analysis but were excluded from all post-hoc analyses pertaining to phenology. Bolded rows indicate statistical significance.
(PDF)

S2 Table Species with unresolved larval diet category. All were included in the GLMM analysis and phenology post-hoc analyses (if they qualified) but excluded from post-hoc larval diet breadth analysis. All conjectures on larval diet are based on related species. Bolded rows indicate statistical significance.
(PDF)

S3 Table Summary table of species included in this study. Higher taxonomic designations, sample size, life history categories, and phenological response curve statistics are included for each. Bolded rows indicate statistical significance.
(PDF)
