# Supplementary material for: Phenological responses of 215 moth species to interannual climate variation in the Pacific Northwest from 1895 through 2013
Source: PLoS One. 2018 Sep 12;13(9):e0202850. doi: 10.1371/journal.pone.0202850 (PMC6135373; doi:10.1371/journal.pone.0202850)
Supplement: S1 Table — All species were classified as having a "mid season" phenology for GLMM analysis but were excluded from all post-hoc analyses pertaining to phenology. Bolded rows indicate statistical significance. (PDF) [file pone.0202850.s001.pdf]

Species	True Phenology	Activity Period (months)	Intercept	Slope	Sensitivity (Days/˚C)
Abagrotis scopeops	mid/late	June - Sept.	5.2853	-0.004034	-0.79
Aseptis characta	early/mid	May - Sept.	5.184549	-0.009966	-1.77
Idia americalis	mid/late	May - Oct.	5.325754	-0.0039	-0.80
Protolampra rufipectus	**mid/late**	**June - Oct.**	**5.41076**	**-0.0281**	**-6.20**
Syngrapha celsa	**mid/late**	**June - Oct.**	**5.388332**	**-0.021972**	**-4.76**

P-value
0.763
0.517
0.635
**0.0095**
**0.00329**
