# Supplementary material for: Phenological responses of 215 moth species to interannual climate variation in the Pacific Northwest from 1895 through 2013
Source: PLoS One. 2018 Sep 12;13(9):e0202850. doi: 10.1371/journal.pone.0202850 (PMC6135373; doi:10.1371/journal.pone.0202850)
Supplement: S2 Table — All were included in the GLMM analysis and phenology post-hoc analyses (if they qualified) but excluded from post-hoc larval diet breadth analysis. All conjectures on larval diet are based on related species. Bolded rows indicate statistical significance. (PDF) [file pone.0202850.s002.pdf]

Species	Data on Larval Diet	Intercept	Slope
Euthyatira semicircularis	Unknown	5.142	0.005
Spilosoma pteridis	**No data. Likely herbaceous sp.**	**5.030**	**-0.019**
Drasteria adumbrata	Unknown	5.178	-0.013
Melipotis jucunda	Unresolved. Willows or herbacious legumes.	5.196	-0.020
Idia americalis	Lichens, dead leaves.	5.326	-0.004
Lygephila victoria	Unresolved. Lab specimens feed on *Lupinus sp.*	5.340	-0.001
Acronicta parallela	Unknown	5.293	-0.027
Acronicta strigulata	No data. Likely monophagous.	5.347	0.007
Cryphia cuerva	No data. Likely rock lichens.	5.460	0.006
Cucullia antipoda	No data. Likely *Asteraceae sp.*	5.083	-0.016
Cucullia strigata	No data. Likely *Asteraceae sp.*	**4.610**	**-0.054**
Abagrotis scopeops	No data. Likely hardwoods.	5.285	-0.004
Apamea antennata	**No data. Likely soil-surface feeder on *Poaceae sp.***	**5.249**	**-0.024**
Apamea cinefacta	**No data. Likely soil-surface feeder on *Poaceae sp.***	4.987	-0.011
Apamea sora	**No data. Likely soil-surface feeder on *Poaceae sp.***	**5.317**	**-0.025**
Apamea spaldingi	No data. Likely soil-surface feeder on sp. in Poaceae.	4.982	-0.007
Aseptis adnixa	Unknown	5.252	0.002
Caradrina meralis	No data. Likely herbaceous genera.	5.441	0.010
Epidemas obscurus	Unknown	5.578	-0.001
Euxoa atomaris	No data. Likely herbaceous sp.	5.519	0.003
Euxoa declarata	No data. Likely herbaceous sp.	5.468	-0.012
Euxoa divergens	No data. Likely herbaceous sp.	5.288	-0.011
Euxoa intrita	No data. Likely herbaceous sp.	5.431	-0.001
Euxoa plagigera	**No data. Likely herbaceous sp.**	**5.366**	**-0.015**
Euxoa satis	No data. Likely herbaceous sp.	5.307	0.006
Euxoa septentrionalis	No data. Likely herbaceous sp.	5.464	0.006
Euxoa terrenus	No data. Likely herbaceous sp.	5.239	-0.003
Lacinipolia cuneata	No data. Likely polyphagous with shrubs and herbs.	5.114	-0.011
Lacinipolia davena	**No data. Likely herbaceous sp.**	**5.256**	**-0.017**
Properigea albimacula	**No data. Likely herbaceous sp.**	**5.320**	**-0.023**
Pseudanarta crocea	No data. Likely oligophagous on *Poaceae sp.*	5.554	0.001
Sunira decipiens	No data. Likely herbaceous sp.	5.608	-0.002

Oligocentria pallida	**No data. Likely hardwoods.**	**5.312**	**-0.026**

Sensitivity (Days/˚C)	P-value
0.89	0.50400
**-2.90**	**0.02800**
-2.28	0.09480
-3.51	0.19600
-0.80	0.63500
-0.20	0.90000
-5.22	0.28500
1.39	0.67100
1.37	0.57600
-2.63	0.11500
**-5.24**	**0.00323**
-0.79	0.76300
**-4.49**	**0.00024**
-1.57	0.20900
**-4.99**	**0.03860**
-0.97	0.64700
0.30	0.86900
2.40	0.13300
-0.18	0.90300
0.79	0.64000
-2.87	0.31200
-2.16	0.14600
-0.33	0.82900
**-3.24**	**0.03200**
1.20	0.41400
1.32	0.39700
-0.63	0.74300
-1.82	0.16600
**-3.29**	**0.04430**
**-4.73**	**0.01290**
0.20	0.90000
-0.62	0.64400

	**-5.28**	**0.00028**
