# Supplementary material for: Phenological responses of 215 moth species to interannual climate variation in the Pacific Northwest from 1895 through 2013
Source: PLoS One. 2018 Sep 12;13(9):e0202850. doi: 10.1371/journal.pone.0202850 (PMC6135373; doi:10.1371/journal.pone.0202850)
Supplement: S3 Table — Higher taxonomic designations, sample size, life history categories, and phenological response curve statistics are included for each. Bolded rows indicate statistical significance. (PDF) [file pone.0202850.s003.pdf]

Family	Subfamily	Tribe	Species	Adult Seasonality	Larval Diet Breadth	Num. Records	Intercept	Slope	Sensitivity Days/˚C	P-value
**Noctuidae**	**Noctuinae**	**Apameini**	***Apamea alia*** **(Guenée, 1852)**	**Mid season**	**Oligophagous**	**98**	**5.180**	**-0.023**	**-4.09**	**0.0157**
**Noctuidae**	**Noctuinae**	**Apameini**	***Apamea amputatrix*** **(Fitch, 1857)**	**Mid season**	**Oligophagous**	**373**	**5.296**	**-0.011**	**-2.20**	**0.0213**
**Noctuidae**	**Noctuinae**	**Apameini**	***Apamea antennata*** **(Smith, 1891)**	**Mid season**		**187**	**5.249**	**-0.024**	**-4.49**	**0.000239**
Noctuidae	Noctuinae	Apameini	*Apamea cinefacta* (Grote, 1881)	Early season		137	4.987	-0.011	-1.57	0.209
**Noctuidae**	**Noctuinae**	**Apameini**	***Apamea cogitata*** **(Smith, 1891)**	**Mid season**	**Oligophagous**	**329**	**5.332**	**-0.017**	**-3.50**	**0.000219**
Noctuidae	Noctuinae	Apameini	*Apamea cuculliformis* (Grote, 1875)	Early season	Oligophagous	82	5.082	0.000	-0.02	0.99
**Noctuidae**	**Noctuinae**	**Apameini**	***Apamea devastator*** **(Brace, 1819)**	**Mid season**	**Oligophagous**	**361**	**5.238**	**-0.018**	**-3.44**	**3.76E-5**
**Noctuidae**	**Noctuinae**	**Apameini**	***Apamea occidens*** **(Grote, 1878)**	**Mid season**	**Oligophagous**	**137**	**5.273**	**-0.037**	**-7.02**	**6.95E-6**
**Noctuidae**	**Noctuinae**	**Apameini**	***Apamea scoparia*** **Mikkola, Mustelin, & Lafontaine, 2000**	**Mid season**	**Oligophagous**	**121**	**5.327**	**-0.019**	**-3.80**	**0.0356**
**Noctuidae**	**Noctuinae**	**Apameini**	***Apamea sora*** **(Smith, 1903)**	**Mid season**		**90**	**5.317**	**-0.025**	**-4.99**	**0.0386**
**Noctuidae**	**Noctuinae**	**Apameini**	***Apamea sordens*** **(Hufnagel, 1766)**	**Early season**	**Oligophagous**	**112**	**5.087**	**-0.022**	**-3.58**	**0.00842**
Noctuidae	Noctuinae	Apameini	*Apamea spaldingi* (Smith, 1909)	Mid season		95	4.982	-0.007	-0.97	0.647
**Noctuidae**	**Noctuinae**	**Apameini**	***Apamea vultuosa*** **(Grote. 1875)**	**Mid season**	**Oligophagous**	**77**	**5.112**	**-0.054**	**-8.71**	**7.76E-5**
Noctuidae	Noctuinae	Apameini	*Helotropha reniformis* (Grote, 1874)	Late season	Monophagous	81	5.430	0.002	0.39	0.869
Noctuidae	Noctuinae	Apameini	*Oligia divesta* (Grote, 1874)	Late season	Oligophagous	198	5.437	-0.012	-2.65	0.0503
Noctuidae	Noctuinae	Apameini	*Resapamea passer* (Guenée, 1852)	Mid season	Oligophagous	76	5.268	-0.003	-0.58	0.796
Noctuidae	Noctuinae	Apameini	*Xylomoia indirecta* (Grote, 1875)	Mid season	Polyphagous	216	5.320	-0.005	-1.11	0.309
**Erebidae**	**Arctiinae**	**Arciini**	***Arctia caja*** **(Linnaeus, 1758)**	**Mid season**	**Polyphagous**	**204**	**5.358**	**-0.012**	**-2.52**	**0.00922**
Erebidae	Arctiinae	Arciini	*Cycnia oregonensis* (Stretch, 1873)	Mid season	Monophagous	115	5.190	-0.018	-3.15	0.16
Erebidae	Arctiinae	Arciini	*Estigmene acrea* (Drury, 1773)	Mid season	Polyphagous	94	5.092	-0.021	-3.42	0.0715
**Erebidae**	**Arctiinae**	**Arciini**	***Gnophaela vermiculata*** **(Grote, 1864)**	**Mid season**	**Monophagous**	**120**	**5.336**	**-0.016**	**-3.23**	**0.0344**
Erebidae	Arctiinae	Arciini	*Grammia nevadensis* (Grote & Robinson, 1866)	Late season	Polyphagous	198	5.450	-0.001	-0.30	0.868
**Erebidae**	**Arctiinae**	**Arciini**	***Grammia ornata*** **(Packard, 1864)**	**Early season**	**Polyphagous**	**350**	**5.067**	**-0.017**	**-2.63**	**0.0182**
Erebidae	Arctiinae	Arciini	*Hyphantria cunea* (Drury, 1773)	Mid season	Polyphagous	143	5.107	0.006	1.07	0.561
**Erebidae**	**Arctiinae**	**Arciini**	***Leptarctia californiae*** **(Walker, 1855)**	**Early season**	**Polyphagous**	**190**	**4.871**	**-0.028**	**-3.54**	**0.00128**
Erebidae	Arctiinae	Arciini	*Lophocampa argentata* (Packard, 1864)	Mid season	Oligophagous	138	5.336	-0.011	-2.19	0.161

**Erebidae**	**Arctiinae**	**Arciini**	***Lophocampa maculata* (Harris, 1841)**	**Mid season**	**Polyphagous**	**389**	**5.175**	**-0.015**	**-2.67**	**0.00488**
Erebidae	Arctiinae	Arciini	*Platarctia parthenos* (Harris, 1850)	Mid season	Polyphagous	99	5.216	-0.004	-0.64	0.663
Erebidae	Arctiinae	Arciini	*Platyprepia virginalis* (Biosduval, 1852)	Mid season	Polyphagous	121	5.162	0.000	0.04	0.974
**Erebidae**	**Arctiinae**	**Arciini**	***Pyrrharctia isabella* (J.E. Smith, 1797)**	**Mid season**	**Polyphagous**	**209**	**5.163**	**-0.025**	**-4.36**	**0.00189**
**Erebidae**	**Arctiinae**	**Arciini**	***Spilosoma pteridis* Hy. Edwards, 1874**	**Early season**		**132**	**5.030**	**-0.019**	**-2.90**	**0.028**
Erebidae	Arctiinae	Arciini	*Spilosoma virginica* Fabricious, 1798	Mid season	Polyphagous	344	5.129	0.007	1.11	0.281
Erebidae	Arctiinae	Arciini	*Tyria jacobaeae* (Linnaeus, 1758)	Early season	Monophagous	94	5.059	-0.022	-3.40	0.0698
Erebidae	Arctiinae	Arctiini	*Spilosoma vagans* (Boisduval, 1852)	Early season	Polyphagous	223	5.074	-0.002	-0.24	0.809
**Erebidae**	**Arctiinae**	**Arctiini**	***Spilosoma vestalis* Packard, 1864**	**Early season**	**Polyphagous**	**112**	**5.048**	**-0.034**	**-5.27**	**0.000476**
**Noctuidae**	**Noctuinae**	**Arzamini**	***Bellura obliqua* (Walker, 1865)**	**Mid season**	**Polyphagous**	**78**	**5.150**	**-0.030**	**-5.09**	**0.0149**
Noctuidae	Noctuinae	Caradrinini	*Caradrina meralis* (Morrison, 1875)	Late season		122	5.441	0.010	2.40	0.133
**Noctuidae**	**Noctuinae**	**Caradrinini**	***Caradrina morpheus* (Hufnagel, 1766)**	**Mid season**	**Polyphagous**	**112**	**5.292**	**-0.046**	**-9.00**	**3.78E-5**
**Erebidae**	**Erebinae**	**Caradrinini**	***Catocala relicta* Walker, [1858]**	**Late season**	**Oligophagous**	**85**	**5.527**	**-0.021**	**-5.19**	**0.0128**
**Erebidae**	**Erebinae**	**Caradrinini**	***Catocala semirelicta* Grote, 1874**	**Late season**	**Oligophagous**	**85**	**5.460**	**0.013**	**3.05**	**0.0296**
Noctuidae	Noctuinae	Caradrinini	*Proxenus miranda* Barnes & McDunnough, 1913	Mid season	Polyphagous	100	5.202	-0.002	-0.43	0.796
**Noctuidae**	**Noctuinae**	**Eriopygini**	***Homorthodes communis* (Dyar, 1904)**	**Late season**	**Monophagous**	**167**	**5.405**	**0.019**	**4.20**	**0.0135**
**Noctuidae**	**Noctuinae**	**Eriopygini**	***Homorthodes furfurata* (Grote, 1875)**	**Mid season**	**Oligophagous**	**92**	**5.318**	**-0.024**	**-4.85**	**0.0398**
Noctuidae	Noctuinae	Eriopygini	*Lacinipolia comis* (Grote, 1876)	Mid season	Polyphagous	189	5.372	-0.002	-0.37	0.805
Noctuidae	Noctuinae	Eriopygini	*Lacinipolia cuneata* (Grote, 1873)	Mid season		172	5.114	-0.011	-1.82	0.166
**Noctuidae**	**Noctuinae**	**Eriopygini**	***Lacinipolia davena* (Smith, 1901)**	**Mid season**		**153**	**5.256**	**-0.017**	**-3.29**	**0.0443**
Noctuidae	Noctuinae	Eriopygini	*Lacinipolia olivacea* (Morrison, 1874)	Mid season	Polyphagous	170	5.359	-0.001	-0.25	0.859
**Noctuidae**	**Noctuinae**	**Eriopygini**	***Lacinipolia patalis* (Grote, 1873)**	**Mid season**	**Oligophagous**	**96**	**5.121**	**-0.037**	**-6.14**	**0.0172**
Noctuidae	Noctuinae	Eriopygini	*Lacinipolia pensilis* (Grote, 1874)	Late season	Polyphagous	244	5.419	-0.002	-0.41	0.796
Noctuidae	Noctuinae	Eriopygini	*Lacinipolia stricta (Walker, 1865)*	Late season	Polyphagous	190	5.490	0.003	0.81	0.478
Noctuidae	Noctuinae	Eriopygini	*Lacinipolia strigicollis* (Wallengren, 1860)	Mid season	Polyphagous	164	5.385	-0.015	-3.32	0.0736
Noctuidae	Noctuinae	Eriopygini	*Protorthodes curtica* (Smith, 1890)	Late season	Polyphagous	128	5.510	-0.010	-2.52	0.149
Noctuidae	Noctuinae	Eriopygini	*Pseudorthodes irrorata* (Smith [1888])	Mid season	Polyphagous	215	5.278	0.003	0.49	0.769

**Noctuidae**	**Noctuinae**	**Eriopygini**	***Zosteropoda hirtipes* Grote, 1874**	**Mid season**	**Polyphagous**	**93**	**5.236**	**-0.028**	**-5.15**	**0.00296**
Erebidae	Erebinae	Euclidiini	*Euclidia ardita* Franclemont, 1957	Early season	Oligophagous	142	5.016	0.007	1.00	0.478
Noctuidae	Noctuinae	Hadenini	*Lacanobia subjuncta* (Grote & Robinson, 1868)	Mid season	Polyphagous	129	5.214	-0.012	-2.28	0.258
Noctuidae	Noctuinae	Hadenini	*Melanchra adjuncta* (Guenée, 1852)	Mid season	Polyphagous	94	5.214	-0.004	-0.77	0.74
**Noctuidae**	**Noctuinae**	**Hadenini**	***Polia nimbosa* (Guenée, 1852)**	**Mid season**	**Polyphagous**	**88**	**5.280**	**-0.020**	**-3.88**	**0.0405**
Noctuidae	Noctuinae	Hadenini	*Polia piniae* Buckett & Bauer, 1967	Mid season	Polyphagous	124	5.303	-0.007	-1.34	0.371
**Noctuidae**	**Noctuinae**	**Hadenini**	***Polia purpurissata* (Grote, 1864)**	**Mid season**	**Polyphagous**	**81**	**5.272**	**0.029**	**5.68**	**0.00292**
Noctuidae	Noctuinae	Hadenini	*Spiramater lutra* (Guenée, 1852)	Mid season	Polyphagous	82	5.174	-0.019	-3.29	0.1
**Noctuidae**	**Noctuinae**	**Hadenini**	***Trichordestra liquida* (Grote, 1881)**	**Early season**	**Polyphagous**	**144**	**5.160**	**-0.024**	**-4.16**	**0.00774**
Erebidae	Lymantriinae	Leucaniini	*Leucania dia* (Grote, 1879)	Early season	Oligophagous	164	5.091	-0.001	-0.20	0.885
Noctuidae	Noctuinae	Leucaniini	*Leucania farcta* (Grote, 1881)	Mid season	Oligophagous	302	5.271	-0.004	-0.84	0.518
**Noctuidae**	**Noctuinae**	**Leucaniini**	***Mythimna oxygala* (Grote, 1881)**	**Mid season**	**Polyphagous**	**230**	**5.261**	**-0.019**	**-3.64**	**0.0121**
Erebidae	Lymantriinae	Leucomini	*Leucoma salicis* (Linnaeus, 1758)	Mid season	Oligophagous	169	5.282	0.004	0.72	0.585
**Erebidae**	**Arctiinae**	**Lithosiini**	***Clemensia albata* Packard, 1864**	**Mid season**	**Monophagous**	**138**	**5.334**	**-0.016**	**-3.30**	**0.0335**
Erebidae	Erebinae	Melipotini	*Drasteria adumbrata* (Behr, 1870)	Mid season		216	5.178	-0.013	-2.28	0.0948
Erebidae	Erebinae	Melipotini	*Drasteria divergens* (Behr, 1870)	Mid season	Monophagous	193	5.198	-0.013	-2.39	0.125
**Erebidae**	**Erebinae**	**Melipotini**	***Drasteria ochracea* (Behr, 1870)**	**Mid season**	**Monophagous**	**144**	**5.121**	**-0.033**	**-5.46**	**0.00461**
Erebidae	Erebinae	Melipotini	*Drasteria sabulosa* (Hy. Edwards, 1881)	Mid season	Monophagous	124	5.201	-0.019	-3.32	0.141
Erebidae	Erebinae	Melipotini	*Melipotis jucunda* Walker, [1858]	Mid season		132	5.196	-0.020	-3.51	0.196
**Noctuidae**	**Noctuinae**	**Noctuini**	***Abagrotis apposita* (Grote, 1878)**	**Mid season**	**Polyphagous**	**95**	**5.150**	**0.060**	**10.67**	**0.000214**
**Noctuidae**	**Noctuinae**	**Noctuini**	***Abagrotis duanca* (Smith, 1908)**	**Mid season**	**Monophagous**	**99**	**5.315**	**0.035**	**7.34**	**0.0348**
Noctuidae	Noctuinae	Noctuini	*Abagrotis reedi* Buckett, 1969	Mid season	Polyphagous	99	5.238	0.007	1.40	0.571
Noctuidae	Noctuinae	Noctuini	*Abagrotis scopeops* (Dyar, 1904)	Mid season		92	5.285	-0.004	-0.79	0.763
Noctuidae	Noctuinae	Noctuini	*Abagrotis trigona* (Smtih, 1893)	Late season	Polyphagous	90	5.216	-0.004	-0.82	0.738
**Noctuidae**	**Noctuinae**	**Noctuini**	***Agrotis vancouverensis* Grote, 1873**	**Mid season**	**Polyphagous**	**208**	**5.203**	**-0.023**	**-4.11**	**0.000227**
Noctuidae	Noctuinae	Noctuini	*Agrotis venerabilis* Walker, [1857]	Late season	Polyphagous	78	5.552	-0.005	-1.26	0.464

Noctuidae	Noctuinae	Noctuini	*Agrotis vetusta* (Walker, 1856)	Late season	Polyphagous	75	5.505	-0.001	-0.18	0.946
Noctuidae	Noctuinae	Noctuini	*Anaplectoides prasina* ([Denis & Schiffermüller], 1775)	Mid season	Polyphagous	140	5.357	0.001	0.20	0.924
Noctuidae	Noctuinae	Noctuini	*Cerastis enigmatica* Lafontaine & Crabo, 1997	Early season	Monophagous	96	4.553	0.002	0.17	0.929
Noctuidae	Noctuinae	Noctuini	*Diarsia esurialis* (Grote, 1881)	Mid season	Polyphagous	144	5.234	0.017	3.26	0.127
Noctuidae	Noctuinae	Noctuini	*Dichagyris variabilis* (Grote, 1874)	Late season	Polyphagous	134	5.475	0.004	0.96	0.437
**Noctuidae**	**Noctuinae**	**Noctuini**	***Eurois astricta* Morrison, 1874**	**Mid season**	**Polyphagous**	**113**	**5.381**	**-0.021**	**-4.60**	**0.0186**
Noctuidae	Noctuinae	Noctuini	*Euxoa albipennis* (Grote, 1876)	Late season	Polyphagous	88	5.526	0.009	2.26	0.195
Noctuidae	Noctuinae	Noctuini	*Euxoa atomaris* (Smith, 1890)	Late season		120	5.519	0.003	0.79	0.64
Noctuidae	Noctuinae	Noctuini	*Euxoa bochus* (Morrison, 1874)	Late season	Polyphagous	95	5.467	-0.007	-1.70	0.345
Noctuidae	Noctuinae	Noctuini	*Euxoa catenula* (Grote, 1879)	Late season	Polyphagous	120	5.536	0.002	0.57	0.683
Noctuidae	Noctuinae	Noctuini	*Euxoa comosa* (Morrison, 1876)	Late season	Polyphagous	113	5.410	0.010	2.33	0.411
Noctuidae	Noctuinae	Noctuini	*Euxoa declarata* (Walker, 1865)	Late season		79	5.468	-0.012	-2.87	0.312
Noctuidae	Noctuinae	Noctuini	*Euxoa divergens* (Walker, [1857])	Mid season		144	5.288	-0.011	-2.16	0.146
Noctuidae	Noctuinae	Noctuini	*Euxoa infausta* (Walker, 1865)	Mid season	Polyphagous	169	5.287	-0.009	-1.73	0.227
Noctuidae	Noctuinae	Noctuini	*Euxoa intrita* (Morrison, 1874)	Late season		77	5.431	-0.001	-0.33	0.829
Noctuidae	Noctuinae	Noctuini	*Euxoa messoria* (Harris, 1841)	Late season	Polyphagous	222	5.465	-0.005	-1.23	0.395
Noctuidae	Noctuinae	Noctuini	*Euxoa olivia* (Morrison, 1876)	Mid season	Polyphagous	135	5.569	-0.006	-1.53	0.295
**Noctuidae**	**Noctuinae**	**Noctuini**	***Euxoa plagigera* (Morrison, 1874)**	**Mid season**		**129**	**5.366**	**-0.015**	**-3.24**	**0.032**
Noctuidae	Noctuinae	Noctuini	*Euxoa satis* (Harvey, 1876)	Mid season		156	5.307	0.006	1.20	0.414
Noctuidae	Noctuinae	Noctuini	*Euxoa septentrionalis* (Walker, 1865)	Late season		153	5.464	0.006	1.32	0.397
Noctuidae	Noctuinae	Noctuini	*Euxoa terrenus* (Smith, 1900)	Mid season		139	5.239	-0.003	-0.63	0.743
Noctuidae	Noctuinae	Noctuini	*Euxoa tessellata* (Harris, 1841)	Mid season	Polyphagous	174	5.311	-0.009	-1.89	0.181
Noctuidae	Noctuinae	Noctuini	*Feltia jaculifera* (Guenée, 1852)	Late season	Polyphagous	183	5.497	0.005	1.17	0.363
Noctuidae	Noctuinae	Noctuini	*Graphiphora augur* (Fabricious, 1775)	Mid season	Polyphagous	110	5.312	-0.009	-1.74	0.327
Noctuidae	Noctuinae	Noctuini	*Ochropleura implecta* Lafontaine, 1998	Mid season	Polyphagous	90	5.239	0.014	2.75	0.286
**Noctuidae**	**Noctuinae**	**Noctuini**	***Protolampra rufipectua* (Morrison, 1875)**	**Mid season**	**Oligophagous**	**85**	**5.411**	**-0.028**	**-6.20**	**0.0095**

**Noctuidae**	**Noctuinae**	**Noctuini**	***Setagrotis pallidicollis* (Grote, 1880)**	**Mid season**	**Polyphagous**	**107**	**5.352**	**-0.023**	**-4.70**	**0.00719**
**Noctuidae**	**Noctuinae**	**Noctuini**	***Spaelotis bicava* Lafontaine, 1998**	**Mid season**	**Polyphagous**	**183**	**5.285**	**-0.025**	**-4.96**	**0.0193**
**Noctuidae**	**Noctuinae**	**Noctuini**	***Spaelotis clandestina* (Harris, 1841)**	**Mid season**	**Polyphagous**	**85**	**5.310**	**-0.052**	**-10.33**	**0.000494**
Noctuidae	Noctuinae	Noctuini	*Xestia infimatis* (Grote, 1880)	Late season	Polyphagous	94	5.514	0.002	0.48	0.764
**Noctuidae**	**Noctuinae**	**Noctuini**	***Xestia oblata* (Morrison, 1875)**	**Mid season**	**Polyphagous**	**124**	**5.310**	**-0.039**	**-7.72**	**9.97E-6**
Noctuidae	Noctuinae	Noctuini	*Xestia smithii* (Snellen, 1896)	Late season	Polyphagous	124	5.436	-0.001	-0.25	0.875
Noctuidae	Noctuinae	Noctuini	*Xestia xanthographa* ([Denis & Schiffermüller], 1775)	Late season	Polyphagous	139	5.490	-0.012	-2.91	0.0626
Erebidae	Erebinae	Omopterini	*Zale minerea* Guenée, 1852	Early season	Polyphagous	114	4.963	-0.005	-0.70	0.675
Erebidae	Lymantriinae	Orgyiini	*Dasychira grisefacta* (Dyar, 1911)	Mid season	Oligophagous	114	5.305	0.004	0.86	0.589
**Erebidae**	**Lymantriinae**	**Orgyiini**	***Dasychira vagans* (Barnes & McDunnough, 1913)**	**Mid season**	**Polyphagous**	**111**	**5.311**	**-0.018**	**-3.65**	**0.0309**
**Erebidae**	**Lymantriinae**	**Orgyiini**	***Orgyia antiqua* (Linnaeus, 1758)**	**Late season**	**Polyphagous**	**75**	**5.481**	**-0.023**	**-5.43**	**0.0178**
Erebidae	Lymantriinae	Orgyiini	*Orgyia pseudostugata* (McDunnough, 1921)	Late season	Oligophagous	89	5.487	-0.012	-2.77	0.186
**Noctuidae**	**Noctuinae**	**Orthosiini**	***Acerra normalis* Grote, 1874**	**Early season**	**Oligophagous**	**119**	**5.158**	**0.037**	**6.54**	**0.0026**
**Noctuidae**	**Noctuinae**	**Orthosiini**	***Egira crucialis* (Harvey, 1875)**	**Early season**	**Polyphagous**	**183**	**4.691**	**-0.031**	**-3.33**	**0.00594**
Noctuidae	Noctuinae	Orthosiini	*Egira curialis* (Harvey, 1875)	Early season	Polyphagous	156	4.686	-0.002	-0.26	0.807
**Noctuidae**	**Noctuinae**	**Orthosiini**	***Egira hiemalis* (Grote, 1874)**	**Early season**	**Polyphagous**	**103**	**4.253**	**-0.085**	**-5.74**	**1.98E-6**
Noctuidae	Noctuinae	Orthosiini	*Egira perlubens* (Grote, 1881)	Early season	Polyphagous	142	4.805	-0.012	-1.45	0.433
Noctuidae	Noctuinae	Orthosiini	*Egira rubrica* (Harvey, 1878)	Early season	Polyphagous	206	4.763	-0.005	-0.56	0.546
Noctuidae	Noctuinae	Orthosiini	*Egira simplex* (Walker, 1865)	Early season	Oligophagous	104	4.739	-0.022	-2.54	0.163
**Noctuidae**	**Noctuinae**	**Orthosiini**	***Orthosia hibisci* (Guenée, 1852)**	**Early season**	**Polyphagous**	**237**	**4.671**	**-0.048**	**-5.01**	**3.40E-7**
Noctuidae	Noctuinae	Orthosiini	*Orthosia praeses* (Grote, 1879)	Early season	Polyphagous	111	4.341	-0.036	-2.73	0.0736
**Noctuidae**	**Noctuinae**	**Orthosiini**	***Orthosia transparens* Grote, 1882**	**Early season**	**Oligophagous**	**96**	**4.741**	**-0.065**	**-7.25**	**5.59E-5**
Noctuidae	Noctuinae	Phlogophorini	*Euplexia benesimilis* McDunnough, 1922	Mid season	Polyphagous	122	5.215	0.000	0.07	0.972
Noctuidae	Noctuinae	Phlogophorini	*Phlogophora periculosa* Guenée, 1852	Late season	Polyphagous	112	5.383	-0.006	-1.31	0.6
Noctuidae	Noctuinae	Tholerini	*Nephelodes minians* Guenée, 1852	Late season	Oligophagous	79	5.506	-0.013	-3.12	0.0756

Noctuidae	Noctuinae	Xylenini	*Agrochola pulchella* (Smith, 1900)	Late season	Polyphagous	107	5.595	-0.008	-2.20	0.227
**Noctuidae**	**Noctuinae**	**Xylenini**	***Agrochola purpurea* (Grote, 1874)**	**Late season**	**Polyphagous**	**135**	**5.552**	**0.029**	**7.47**	**0.000948**
**Noctuidae**	**Noctuinae**	**Xylenini**	***Andropolia diversilineata* (Grote, 1877)**	**Late season**	**Oligophagous**	**84**	**5.395**	**-0.045**	**-9.68**	**0.000483**
Noctuidae	Noctuinae	Xylenini	*Aseptis adnixa* (Grote, 1880)	Mid season		154	5.252	0.002	0.30	0.869
**Noctuidae**	**Noctuinae**	**Xylenini**	***Aseptis binotata* (Walter, 1865)**	**Mid season**	**Polyphagous**	**254**	**5.331**	**-0.023**	**-4.65**	**0.000354**
Noctuidae	Noctuinae	Xylenini	*Aseptis characta* (Grote, 1880)	Mid season	Monophagous	120	5.185	-0.010	-1.77	0.517
**Noctuidae**	**Noctuinae**	**Xylenini**	***Aseptis fumosa* (Grote, 1879)**	**Mid season**	**Polyphagous**	**95**	**5.279**	**-0.044**	**-8.50**	**9.57E-5**
Noctuidae	Noctuinae	Xylenini	*Cosmia praeacuta* (Smith, 1894)	Late season		180	5.426	-0.006	-1.31	0.46
Noctuidae	Noctuinae	Xylenini	*Epidemas obscurus* Smith, 1903	Late season		140	5.578	-0.001	-0.18	0.903
Noctuidae	Noctuinae	Xylenini	*Fishia discors* (Grote, 1881)	Late season	Polyphagous	161	5.588	-0.002	-0.48	0.714
Noctuidae	Noctuinae	Xylenini	*Fishia illocata* (Walker, 1857)	Late season	Polyphagous	131	5.527	-0.011	-2.81	0.0576
Noctuidae	Noctuinae	Xylenini	*Mesogona olivata* (Harvey, 1874)	Late season	Polyphagous	193	5.545	0.002	0.62	0.587
**Noctuidae**	**Noctuinae**	**Xylenini**	***Platypolia contadina* (Smith, 1894)**	**Late season**	**Polyphagous**	**136**	**5.500**	**0.019**	**4.76**	**0.0459**
**Noctuidae**	**Noctuinae**	**Xylenini**	***Properigea albimacula* (Barnes & McDunnough, 1912)**	**Mid season**		**99**	**5.320**	**-0.023**	**-4.73**	**0.0129**
Noctuidae	Noctuinae	Xylenini	*Pseudanarta crocea* (H. Edwards, 1875)	Late season		101	5.554	0.001	0.20	0.9
Noctuidae	Noctuinae	Xylenini	*Sunira decipiens* (Grote, 1881)	Late season		142	5.608	-0.002	-0.62	0.644
Noctuidae	Noctuinae	Xylenini	*Zotheca tranquilla* Grote, 1874	Mid season	Monophagous	187	5.334	-0.001	-0.29	0.845
Noctuidae	Acronictinae		*Acronicta dactylina* Grote, 1874	Mid season	Polyphagous	224	5.149	-0.010	-1.66	0.334
Noctuidae	Acronictinae		*Acronicta grisea* Walker, 1856	Mid season	Polyphagous	160	5.251	-0.004	-0.83	0.659
Noctuidae	Acronictinae		*Acronicta impleta* Walker, 1856	Early season	Polyphagous	94	5.294	-0.018	-3.61	0.0906
Noctuidae	Acronictinae		*Acronicta innotata* Guenée, 1852	Mid season	Polyphagous	96	5.135	-0.005	-0.88	0.785
**Noctuidae**	**Acronictinae**		***Acronicta marmorata* Smith, 1897**	**Mid season**	**Monophagous**	**99**	**5.164**	**-0.034**	**-5.79**	**0.0178**
Noctuidae	Acronictinae		*Acronicta parallela* (Grote, 1879)	Mid season		84	5.293	-0.027	-5.22	0.285
Noctuidae	Acronictinae		*Acronicta perdita* Grote, 1874	Early season	Polyphagous	109	5.204	0.018	3.23	0.182
Noctuidae	Acronictinae		*Acronicta radcliffei* (Harvey, 1875)	Mid season	Oligophagous	113	5.276	-0.019	-3.77	0.137
Noctuidae	Acronictinae		*Acronicta strigulata* Smith, 1897	Mid season		82	5.347	0.007	1.39	0.671

Noctuidae	Amphipyrinae	*Amphipyra pyramidoides* Guenée, 1852	Late season	Polyphagous	119	5.459	-0.002	-0.50	0.804
Noctuidae	Amphipyrinae	*Amphipyra tragopoginis* (Clerck, 1759)	Mid season	Polyphagous	109	5.414	-0.011	-2.51	0.3
Noctuidae	Plusiinae	*Anagrapha falcifera* (W. Kriby, 1837)	Mid season	Polyphagous	115	5.267	-0.031	-5.84	0.0785
**Saturniidae**	**Saturniinae**	***Antheraea polyphemus* (Cramer, 1776)**	**Mid season**	**Polyphagous**	**224**	**5.152**	**-0.016**	**-2.79**	**0.0386**
**Noctuidae**	**Plusiinae**	***Autographa ampla* (Walker, [1858])**	**Mid season**	**Polyphagous**	**172**	**5.307**	**-0.017**	**-3.50**	**0.0129**
Noctuidae	Plusiinae	*Autographa corusca* (Strecker, 1885)	Mid season	Monophagous	105	5.362	-0.010	-2.18	0.258
**Noctuidae**	**Plusiinae**	***Autographa mappa* (Grote & Robinson, 1868)**	**Mid season**	**Polyphagous**	**78**	**5.243**	**-0.035**	**-6.57**	**0.00274**
**Noctuidae**	**Oncocnemidinae**	***Behrensia conchiformis* Grote, 1875**	**Early season**	**Oligophagous**	**172**	**4.770**	**-0.064**	**-7.28**	**1.2E-7**
Erebidae	Herminiinae	*Bleptina caradrinalis* Guenée, 1854	Mid season	Polyphagous	89	5.237	-0.009	-1.67	0.42
**Uraniidae**	**Epipleminae**	***Callizzia amorata* Packard, 1876**	**Mid season**	**Oligophagous**	**147**	**5.253**	**-0.015**	**-2.91**	**0.0153**
Drepenidae	Thyatirinae	*Ceranemota fasciata* (Barnes & McDunnough)	Late season	Oligophagous	79	5.583	-0.001	-0.34	0.87
Drepenidae	Thyatirinae	*Ceranemota tearlei* (Hy. Edwards, 1886)	Late season	Oligophagous	93	5.532	0.018	4.59	0.0795
**Erebidae**	**Arctiinae**	***Cisseps fulvicollis* (Hüber, 1818)**	**Mid season**	**Polyphagous**	**125**	**5.260**	**-0.024**	**-4.62**	**0.0142**
**Notodontidae**	**Pygaerinae**	***Clostera apicalis* (Walker, 1855)**	**Mid season**	**Oligophagous**	**213**	**5.120**	**-0.015**	**-2.53**	**0.0428**
Noctuidae	Bryophilinae	*Cryphia cuerva* (Barnes, 1907)	Late season		98	5.460	0.006	1.37	0.576
Noctuidae	Cuculliinae	*Cucullia antipoda* Strecker, 1878	Early season		144	5.083	-0.016	-2.63	0.115
**Noctuidae**	**Cuculliinae**	***Cucullia strigata* (Smith, 1892)**	**Early season**		**79**	**4.610**	**-0.054**	**-5.24**	**0.00323**
Drepenidae	Thyatirinae	*Euthyatira semicircularis* (Grote)	Mid season		144	5.142	0.005	0.89	0.504
**Noctuidae**	**Amphipyrinae**	***Feralia comstocki* Grote, 1874**	**Early season**	**Oligophagous**	**91**	**4.808**	**-0.025**	**-2.98**	**0.0295**
Noctuidae	Amphipyrinae	*Feralia deceptiva* McDunnough, 1920	Early season	Monophagous	99	4.554	0.001	0.05	0.969
**Notodontidae**	**Notodontinae**	***Furcula cinerea* (Walker, 1865)**	**Mid season**	**Oligophagous**	**78**	**5.200**	**-0.032**	**-5.71**	**0.0296**
**Notodontidae**	**Notodontinae**	***Furcula scolopendrina* (Boisduval, 1869)**	**Mid season**	**Polyphagous**	**209**	**5.201**	**-0.018**	**-3.18**	**0.0197**
Notodontidae	Notodontinae	*Gluphisia septentrionis* Walker, 1855	Mid season	Oligophagous	145	5.178	0.012	2.18	0.101
Notodontidae	Notodontinae	*Gluphisia severa* Hy. Edwards, 1866	Early season	Oligophagous	192	4.830	-0.008	-0.98	0.435
**Drepenidae**	**Thyatirinae**	***Habrosyne scripta* (Gosse)**	**Mid season**	**Monophagous**	**264**	**5.278**	**-0.025**	**-4.74**	**0.0003**
Noctuidae	Heliothinae	*Helicoverpa zea* (Bodie, 1850)	Late season	Polyphagous	100	5.494	-0.024	-5.71	0.121

**Sphingidae**	**Macroglossinae**	***Hemaris thetis* (Boisduval, 1855)**	**Early season**	**Oligophagous**	**306**	**5.063**	**-0.018**	**-2.74**	**0.00422**
Saturniidae	Hemileucinae	*Hemileuca eglanterina* (Boisduval, 1852)	Mid season	Polyphagous	233	5.273	-0.011	-2.10	0.0593
Saturniidae	Saturniinae	*Hyalophora euryalus* (Boisduval, 1855)	Early season	Polyphagous	187	4.982	-0.010	-1.44	0.313
Sphingidae	Macroglossinae	*Hyles gallii* (Rottemburg, 1775)	Mid season	Monophagous	93	5.226	0.001	0.26	0.904
Erebidae	Herminiinae	*Idia americalis* (Guenée, 1854)	Mid season		200	5.326	-0.004	-0.80	0.635
Erebidae	Toxocampinae	*Lygephila victoria* (Grote, 1874)	Mid season		305	5.340	-0.001	-0.20	0.9
Lasiocampidae	Lasiocampinae	*Malacosoma californicum* (Packard, 1864)	Mid season	Polyphagous	196	5.287	-0.010	-1.99	0.0542
Lasiocampidae	Lasiocampinae	*Malacosoma disstria* (Hüber, 1820)	Mid season	Polyphagous	196	5.301	0.003	0.58	0.584
Notodontidae	Phalerinae	*Nadata gibbosa* (J.E. Smith, 1797)	Mid season	Polyphagous	282	5.201	-0.002	-0.39	0.717
**Notodontidae**	**Heterocampinae**	***Oligocentria pallida* (Strecker, 1899)**	**Mid season**		**128**	**5.312**	**-0.026**	**-5.28**	**0.000281**
**Notodontidae**	**Heterocampinae**	***Oligocentria semirufescens* (Walker, 1865)**	**Mid season**	**Polyphagous**	**198**	**5.308**	**-0.015**	**-2.96**	**0.0123**
Erebidae	Herminiinae	*Palthis angulalis* (Hübner, 1769)	Mid season	Polyphagous	81	5.290	-0.001	-0.30	0.908
**Noctuidae**	**Pantheinae**	***Panthea virginarius* (Grote, 1880)**	**Mid season**	**Oligophagous**	**387**	**5.244**	**-0.019**	**-3.55**	**0.00181**
Sphingidae	Smerinthinae	*Paonias excaecatus* (J.E. Smith, 1797)	Mid season	Polyphagous	151	5.177	-0.014	-2.47	0.086
**Sphingidae**	**Smerinthinae**	***Paonias myops* (J.E. Smith, 1797)**	**Mid season**	**Polyphagous**	**122**	**5.181**	**-0.021**	**-3.63**	**0.0193**
**Lasiocampidae**	**Lasiocampinae**	***Phyllodesma americana* (Harris, 1841)**	**Early season**	**Polyphagous**	**371**	**5.018**	**-0.037**	**-5.53**	**4.37E-6**
Noctuidae	Oncocnemidinae	*Pleromelloida cinerea* (Smith, 1904)	Late season	Oligophagous	148	5.561	0.004	1.02	0.433
Noctuidae	Oncocnemidinae	*Pleromelloida conserta* (Grote, 1881)	Early season	Oligophagous	156	4.637	-0.022	-2.22	0.101
**Sphingidae**	**Macroglossinae**	***Proserpinus clarkiae* (Boisduval, 1852)**	**Mid season**	**Oligophagous**	**141**	**4.954**	**-0.018**	**-2.50**	**0.0298**
**Noctuidae**	**Eustrotiinae**	***Protodeltote albidula* (Guenée, 1852)**	**Mid season**	**Oligophagous**	**167**	**5.185**	**-0.020**	**-3.50**	**0.0254**
**Drepenidae**	**Thyatirinae**	***Pseudothyatira cymatophoroides* Guenée**	**Mid season**	**Polyphagous**	**215**	**5.279**	**-0.019**	**-3.64**	**0.000154**
Noctuidae	Raphiinae	*Raphia frater* Grote, 1864	Mid season	Polyphagous	224	5.207	-0.015	-2.73	0.0528
Noctuidae	Heliothinae	*Schinia acutilinea* (Grote, 1878)	Late season	Monophagous	93	5.482	0.011	2.73	0.195
Notodontidae	Heterocampinae	*Schizura ipomoeae* Doubleday, 1841	Mid season	Polyphagous	183	5.286	0.001	0.21	0.868
Notodontidae	Heterocampinae	*Schizura unicornis* (J.E. Smith, 1797)	Mid season	Polyphagous	193	5.254	-0.011	-2.06	0.105

**Sphingidae**	**Smerinthinae**	***Smerinthus opthalmica* Boisduval, 1855**	**Mid season**	**Oligophagous**	**516**	**5.157**	**-0.011**	**-1.98**	**0.0172**
Sphingidae	Sphinginae	*Sphinx perelegans* Hy. Edwards, 1874	Mid season	Polyphagous	86	5.179	-0.008	-1.41	0.442
Sphingidae	Sphinginae	*Sphinx vashti* Strecker, 1878	Mid season	Monophagous	232	5.119	0.004	0.68	0.546
**Noctuidae**	**Oncocnemidinae**	***Sympistis greyi (*Troughbridge & Crabo, (1999))**	**Mid season**	**Monophagous**	**128**	**5.246**	**-0.023**	**-4.31**	**0.00443**
**Noctuidae**	**Plusiinae**	***Syngrapha celsa* (H. Edwards, 1881)**	**Mid season**	**Oligophagous**	**159**	**5.388**	**-0.022**	**-4.76**	**0.00329**
**Noctuidae**	**Plusiinae**	***Syngrapha orophila* Hampson, 1908**	**Mid season**	**Monophagous**	**97**	**5.358**	**-0.019**	**-3.94**	**0.0171**
Noctuidae	Plusiinae	Syngrapha rectangula (W. Kirby, 1837)	Late season	Oligophagous	116	5.358	-0.003	-0.69	0.691
Lasiocampidae	Macromphaliinae	*Tolype distincta* French, 1890	Late season	Oligophagous	217	5.505	-0.005	-1.16	0.309
